# Association between remnant cholesterol and insulin resistance levels in patients with metabolic-associated fatty liver disease

**DOI:** 10.1038/s41598-024-55282-4

**Published:** 2024-02-26

**Authors:** Shuang Wang, Qiang Zhang, Bo Qin

**Affiliations:** 1 Department of Infectious Diseases, The First Affiliated Hospital of Chongqing Medical University, Chongqing, China; 2 Department of Gastroenterology, The People’s Hospital of Changshou, Chongqing, China

**Keywords:** Metabolic-associated fatty liver disease, Remnant cholesterol, Insulin resistance, HOMA-IR, Liver steatosis, Endocrine system and metabolic diseases, Diagnostic markers

## Abstract

The relationship between remnant cholesterol (RC) and homeostasis model assessment-insulin resistance (HOMA-IR) in the context of metabolic-associated fatty liver disease (MAFLD) remains an area of ambiguity. This investigation was designed to elucidate the potential association between RC and HOMA-IR in a cohort of American adults diagnosed with MAFLD. Data from 5533 participants were procured from the 2017–2018 US National Health and Nutrition Examination (NHANES) databases. A weighted linear regression model was employed to analyze the association between RC and HOMA-IR in the context of MAFLD. Preliminary analysis revealed that 44.67% of the participants were diagnosed with MAFLD, with a higher prevalence observed in individuals aged 50–64 years (31.84%, *p* < 0.0001) and in males compared to females (53.48% vs. 46.52%, *p* < 0.0001). A positive correlation was identified between RC and HOMA-IR in MAFLD patients. The threshold effect analysis model indicated a breakpoint at RC = 30 mg/dl, with a more pronounced positive correlation when RC < 30 mg/dl (β = 0.17, *p* < 0.001). Receiver operating characteristic analysis further demonstrated that among all lipid parameters, RC exhibited the largest area under the curve. The study findings suggest a positive correlation between RC and HOMA-IR in MAFLD patients, indicating that elevated RC may serve as an independent risk factor for MAFLD.

## Introduction

Non-alcoholic fatty liver disease (NAFLD) is a prevalent chronic liver condition with significant implications for patient prognosis, healthcare costs, and public health^1,2^. The recent shift in nomenclature to metabolic dysfunction-associated fatty liver disease (MAFLD) underscores its close ties with metabolic abnormalities^3–5^. MAFLD is diagnosed based on the presence of hepatic steatosis along with metabolic disorders like obesity, diabetes, or metabolic dysregulation in lean individuals, offering a broader diagnostic framework compared to NAFLD^6–8^.

Insulin resistance (IR), a condition where the body's cells exhibit a diminished response to the hormone insulin, plays a pivotal role in a host of metabolic disorders. It is a central feature in the development and progression of metabolic-associated liver diseases, including MAFLD. IR disrupts normal glucose and lipid metabolism, leading to increased fat synthesis and accumulation in the liver, a process known as hepatic steatosis^9,10^. This metabolic imbalance not only contributes to the buildup of fat in the liver but also exacerbates inflammation and fibrosis, which are key factors in the progression of liver disease^11^.

Dyslipidemia, characterized by abnormal lipid levels in the blood, plays a critical role in the pathogenesis of MAFLD. Notably, very low-density lipoproteins (VLDL) and their remnants are key contributors to triglyceride accumulation in metabolic liver disease^12^. Remnant cholesterol (RC), comprising cholesterol in VLDL, intermediate-density lipoproteins, and remnants of postprandial chylomicrons post-lipolysis, emerges as a significant factor in MAFLD. RC acts as a cholesterol-rich lipoprotein that promotes liver fat accumulation. Additionally, RC remnants can penetrate the hepatic endothelium and activate Kupffer cells, triggering local inflammation^13^. Through these pathways, RC may contribute to the development and progression of steatohepatitis. Thus, RC represents an emerging cardiometabolic risk factor that may be implicated directly in MAFLD pathogenesis^14^. This elevation in RC, combined with insulin resistance, which further dysregulates lipid metabolism, leads to an increase in RC and other lipotoxic species^15^. The accumulation of these metabolites in the liver can cause cellular stress and damage, potentially exacerbating MAFLD progression.

While previous research has investigated the roles of RC in NAFLD^16,17^, MAFLD presents a different clinical entity with distinct pathophysiological mechanisms. Unlike NAFLD, MAFLD is diagnosed based on metabolic dysfunctions, irrespective of alcohol consumption or other liver diseases. This distinction underscores the importance of examining the combined effects of RC and IR in MAFLD, particularly in the context of diverse populations. Most existing studies on NAFLD have focused on isolated risk factors or specific populations, leaving a critical need for research on the interplay of these factors in MAFLD. Therefore, this study aims to address this gap by exploring the association between RC and IR levels in a multi-ethnic cohort of MAFLD patients, utilizing data from the 2017–2018 National Health and Nutrition Examination Survey (NHANES).

## Methods

### Study design and participants

This study utilized data from the 2017–2018 cycle of the National Health and Nutrition Examination Survey (NHANES), a comprehensive cross-sectional survey executed by the National Center for Health Statistics of the Centers for Disease Control and Prevention in the United States. Notably, this cycle of NHANES introduced the assessment of liver function using ultrasound and vibration-controlled transient elastography (VCTE) for the first time. Liver steatosis was determined using a median Controlled Attenuation Parameter (CAP), while liver fibrosis was defined based on a median Liver Stiffness Measurement (LSM)^18^. The survey methodology included structured household interviews, which provided self-reported demographic data and medical records. Moreover, physical examinations, encompassing anthropometric measurements and blood sample collection, were conducted at mobile examination centers^19,20^.

### Diagnostic criteria and definition of groups

#### Definition of MAFLD

The diagnosis of MAFLD was established based on the presence of hepatic steatosis, as determined by ultrasound, in conjunction with either excess weight/obesity, diabetes mellitus, or metabolic dysregulation^3^. Metabolic dysfunction was defined as the presence of at least two of the seven metabolic anomalies, as per international consensus: waist circumference ≥ 102 cm in men or 88 cm in women; blood pressure ≥ 130/85 mmHg or anti-hypertensive therapy; triglyceride level ≥ 150 mg/dL or specific medication; high-density lipoprotein cholesterol (HDL-C) level < 40 mg/dL for men and < 50 mg/dL for women; prediabetes was defined as fasting plasma glucose levels of 100–125 mg/dL, or HbA1c levels of 5.7% to 6.4%; HOMA-IR ≥ 2.5; and C-reactive protein (CRP) level > 2 mg/L.

#### Definition of liver steatosis and significant fibrosis

In this study, liver steatosis and clinically significant fibrosis (CSF; fibrosis stage ≥ 2) were defined by CAP and LSM cutoffs^21^. A CAP ≥ 285 dB/m (80% sensitivity and 77% specificity) was defined as hepatic steatosis, whereas LSM ≥ 8.6 kPa (66% sensitivity and 80% specificity) was defined as CSF.

### Smoking and alcohol consumption

Smoking status was categorized as current, forlkmer, or never. Alcohol use was classified as never (< 12 drinks in a lifetime), former (≥ 12 drinks in a year and did not drink last year or did not drink last year but drank ≥ 12 drinks in a lifetime), current light/moderate (average of ≤ 1 drink per day for women and ≤ 2 drinks per day for men during the past year), and current heavy (average of > 1 drink per day for women or > 2 drinks per day for men over the past year) drinker^22^.

### Demographic variables

The demographic data including age (18–34, 35–49, 50–64, ≥ 65 years), sex, race/ethnicity (White, Black, Hispanics/Latin Americans, Asian, other (non-Hispanic, including multiracial persons)), educational attainment (EA) (< high school, high school or General Education Development high school equivalency test (GED), ≥ college), poverty income ratio (PIR; < 1.30, 1.30–3.49, ≥ 3.50), and complication were self-reported in accordance with NHANES design. Body mass index (BMI) was calculated as weight (in kilograms) divided by the square of height (in meters). The Centers for Disease Control defined BMI status as normal weight (BMI ≤ 25.0), overweight (BMI 25.0–30.0), and obese (BMI ≥ 30.0)^23^. Also, ongoing variables, including systolic blood pressure, diastolic blood pressure, waist circumference, white blood cell count, hemoglobin, platelet count, hypersensitive-CRP, aspartate aminotransferase, alanine aminotransferase, gamma-glutamyl transferase, total cholesterol (TC), HDL-C, LDL-C, triglycerides (TG), albumin, plasma fasting glucose, fasting insulin, and HbA1c, were measured in the mobile testing centres using standard protocol . HOMA-IR was used to indicate IR by calculating fasting insulin (μU/mL) × fasting glucose (mmol/L)/22.5. RC = TC–HDL–C–LDL–C. Participants were divided into four groups, categorized into quartiles based on their RC levels. The quartile ranges were specifically defined as follows: Quartile 1 (Q1) (2–12 mg/dL), Quartile 2 (Q2) (13–18 mg/dL), Quartile 3 (Q3) (19–27 mg/dL), and Quartile 4 (Q4) (28- 99 mg/dL).

### Statistical analysis

Given the complex design of NHANES, sampling weights were applied in all statistical analyses to ensure the representativeness of the results. For categorical variables such as age group, sex, race, education level, and comorbidities, percentages were calculated and analyzed using the chi-square test. Continuous variables, including various blood biochemical and physical measurements, were analyzed using t-tests.

To explore the correlation between RC and HOMA-IR within the MAFLD population, both univariate and multivariate linear regression analyses were employed. These analyses were adjusted for confounding variables, including age, race, education, BMI, smoking status, PIR, and alcohol intake status. The weighted linear regression model was specifically chosen to account for the NHANES survey's complex sampling structure, ensuring findings are reflective of the broader U.S. population.

Additionally, a threshold effect analysis model was utilized to determine whether there exists a specific point at which changes in RC levels begin to significantly impact HOMA-IR. This analysis helps in identifying potential nonlinear relationships and interaction effects, providing a deeper understanding of how RC levels influence insulin resistance in the context of MAFLD.

All analyses were conducted using R Studio software (version 4.2.0). A p-value less than 0.05 (two-tailed) was considered statistically significant.

### Ethics approval and consent to participate

NHANES protocol approved by NCHS Research Ethics Review Board, and obtained informed consent from all participants.

## Results

### Prevalence

The prevalence of MAFLD in our study population was found to be 44.67%. Patients diagnosed with MAFLD were predominantly male and older in age. Detailed characteristics of the participants with MAFLD are outlined in Table 1. The highest proportion of MAFLD was observed in participants aged between 50 and 64 years (31.84%, *p* < 0.0001). In contrast, a larger proportion of individuals without MAFLD held a college degree or higher (32.29% vs. 26.57%, *p* < 0.01).

**Table1:** Baseline characteristic of participants.

Characteristics	Non-MAFLD	MAFLD	*P-value*
	n(%)	n(%)	
	n = 3061	n = 2472	
Age			< 0.0001
18–34	1030 (37.46)	436 (20.61)	
35–49	625 (23.39)	544 (24.52)	
50–64	716 (22.11)	790 (31.84)	
> = 65	690 (17.04)	702 (23.03)	
Sex			< 0.0001
Female	1706 (55.93)	1155 (46.52)	
Male	1355 (44.07)	1317 (53.48)	
Ethnicity			< 0.001
White	1017 (61.71)	881 (62.37)	
Black	807 (12.92)	476 (9.38)	
Hispanics/Latin Americans	323 (7.07)	429 (11.61)	
Asian	275 (7.31)	244 (6.67)	
Other	639 (10.98)	442 (9.98)	
Educational level			0.01
Less than High school	611 (11.79)	519 (11.88)	
High school graduate or GED	1709 (55.93)	1433 (61.55)	
Some college or above	741 (32.29)	520 (26.57)	
Poverty income ratio			0.55
< 1.30	927 (21.74)	703 (20.50)	
1.30–3.49	1237 (35.44)	1052 (37.61)	
> = 3.50	897 (42.82)	717 (41.90)	
Smoking			0.01
Never	1889 (60.72)	1412 (56.51)	
Former	593 (21.31)	667 (27.57)	
Current	579 (17.97)	393 (15.92)	
Alcohol			0.04
Never	340 (9.74)	245 (9.55)	
Mild	957 (43.17)	824 (46.63)	
Moderate	510 (24.88)	318 (18.12)	
Heavy	466 (22.21)	414 (25.70)	

n(%) for categorical variables.

### Comorbidities and laboratory and physical parameters

Table 2 presents the prevalence of comorbidities and the distribution of laboratory and physical parameters among the participants. Comorbidities were more prevalent in individuals with MAFLD compared to those without. Liver enzyme levels and metabolic indices were also slightly elevated in participants with MAFLD. The study observed a positive correlation between the levels of RC and HOMA-IR and the prevalence of MAFLD (*p* < 0.0001).

**Table 2:** Laboratory and comorbidities of non-MAFLD and MAFLD.

Characteristics	Non-MAFLD	MAFLD	*P-value*
	n = 2615	n = 2251	
Comorbidities n (%)			
Metabolic disease	340 (10.14)	985 (43.14)	< 0.0001
Cardiovascular disease	212 (5.27)	282 (10.86)	< 0.0001
Cancer	237 (9.40)	250 (11.61)	0.04
Blood and physical measurement			
Body mass index (kg/m^2^)	26.48 (26.08, 26.89)	33.15 (32.56, 33.74)	< 0.0001
Waist circumference (cm)	91.92 (90.91, 92.93)	109.72 (108.26, 111.19)	< 0.0001
Fasting insulin (uU/ml)	11.47 (10.82, 12.13)	17.90 (16.71, 19.09)	< 0.0001
Plasma fasting glucose (mg/dl)	104.53 (103.59, 105.46)	118.79 (116.62, 120.95)	< 0.0001
HbA1c (%)	5.48 (5.45, 5.51)	5.94 (5.89, 5.99)	< 0.0001
Aspartate aminotransferase (U/L)	21.17 (20.45, 21.89)	23.37 (22.03, 24.71)	0.01
Alanine aminotransferase (U/L)	19.43 (18.72, 20.15)	26.35 (25.24, 27.46)	< 0.0001
Gamma–glutamyl transferase (U/L)	24.15 (22.65, 25.65)	35.40 (33.47, 37.34)	< 0.0001
Triglycerides (mg/dl)	151.32 (143.86, 158.79)	193.89 (181.46, 206.31)	< 0.0001
Total cholesterol (mg/dl)	185.35 (182.89, 187.81)	190.59 (186.18, 195.01)	0.01
HDL (mg/dl)	57.17 (56.22, 58.12)	49.71 (48.04, 51.37)	< 0.0001
LDL (mg/dl)	98.59 (96.38, 100.80)	103.01 (99.61, 106.41)	0.01
Albumin (g/dl)	4.10 (4.07, 4.14)	4.05 (4.01, 4.08)	0.01
HS-CRP (mg/l)	3.04 (2.69, 3.39)	5.73 (5.09, 6.36)	< 0.0001
RC (mg/dl)	18.08 (17.14, 19.01)	27.17 (25.12, 29.23)	< 0.0001
RC (quartile)			< 0.0001
2–12	38.09 (33.85, 42.32)	12.99 (10.11, 15.88)	
13–18	26.58 (23.20, 29.96)	20.74 (17.46, 24.02)	
19–27	19.54 (16.86, 22.21)	30.23 (25.91, 34.54)	
28–99	15.80 (13.29, 18.31)	36.04 (31.37, 40.71)	
HOMA-IR	2.53 (2.21, 2.85)	5.71 (5.08, 6.34)	< 0.0001
HOMA-IR (quartile)			< 0.0001
0.12–1.58	478 (41.09)	107 (11.57)	
1.58–2.56	404 (30.73)	182 (18.57)	
2.56–4.59	297 (20.24)	312 (29.86)	
4.59–179.18	131 (7.94)	464 (39.99)	
VCTE measurements			
CAP (dB/m)	221.04 (218.97, 223.12)	315.10 (312.39, 317.80)	< 0.0001
LSM (kPa)	5.15 (5.01, 5.29)	6.81 (6.47, 7.14)	< 0.0001
			< 0.0001
CAP < 285	96.92 (96.04, 97.81)	24.11 (21.01, 27.21)	
CAP ≥ 285/LSM < 8.6	1.91 (1.36, 2.47)	36.54 (33.28, 39.81)	
CAP ≥ 285/LSM ≥ 8.6	1.16 (0.62, 1.70)	39.35 (36.10, 42.59)	

Weighted mean or percentage with 95% confidence intervals for continuous variables.

n(%) for categorical variables.

*HbA1c* glycosylated hemoglobin, type A1c; *HDL* high-density lipoprotein cholesterol; *LDL* low-density lipoprotein cholesterol; *RC* remnant cholesterol; *HS-CRP* hypersensitive-c-reactive-protein; *HOMA-IR* homeostasis model assessment-insulin resistance; *CAP* controlled attenuation parameter; *LSM* liver stiffness measure; *VCTE* vibration- controlled transient elastography.

### Relationships among RC, MAFLD, and HOMA-IR

**Figure 1:**
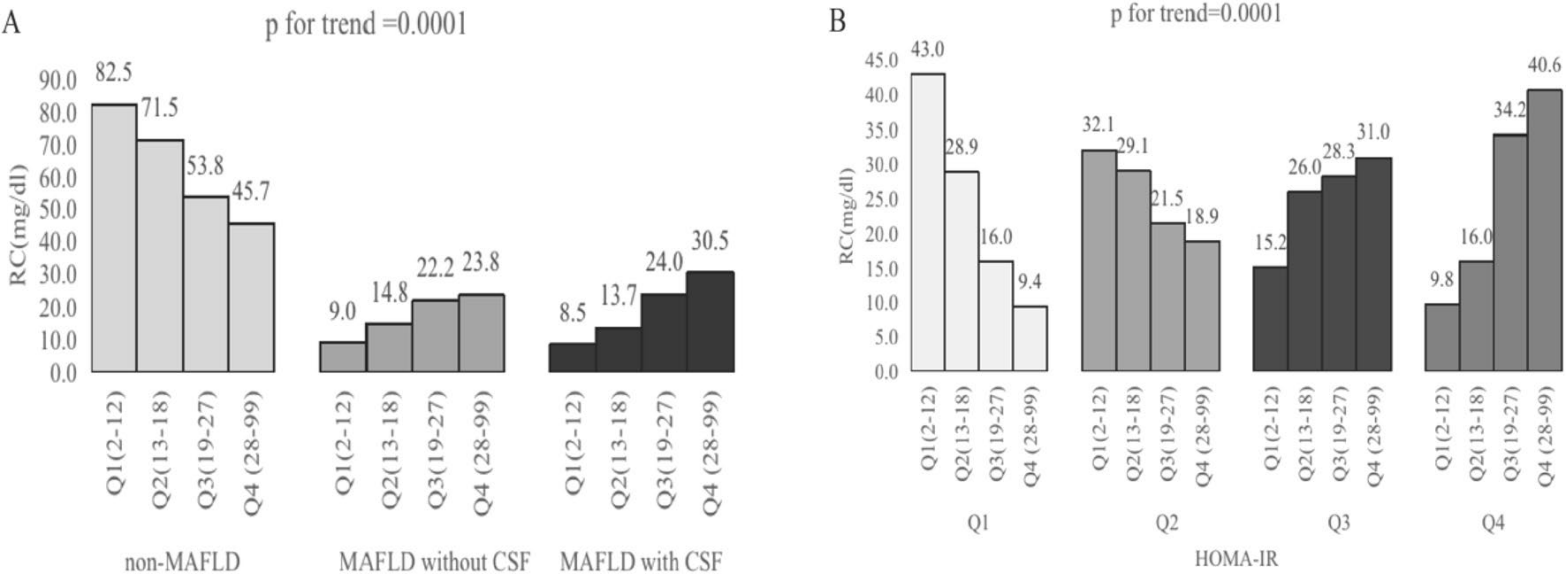
The prevalence of remnant cholesterol (RC) quartiles by MAFLD phenotypes and HOMA-IR quartiles. (**A**) MAFLD phenotypes (non-MAFLD, MAFLD without clinical significant fibrosis (CSF), MAFLD with clinical significant fibrosis (CSF). (**B**) HOMA-IR quartiles (0.12–1.58, 1.58–2.56, 2.56–4.59, 4.59–179.18).

Tables 3 and 4 display the baseline characteristics of the participants and the physical and laboratory parameters across RC quartiles, respectively. Significant differences were observed across RC quartiles in terms of age, ethnicity, educational level, BMI, waist circumference, blood pressure, liver enzymes, routine blood, and metabolic-related index distribution. RC levels were positively associated with the prevalence of MAFLD and HOMA-IR in a dose-dependent manner (*p* for trend = 0.0001, Fig. 1A,B). As shown in Table 5, there was a positive correlation between RC and HOMA-IR (odds ratio (OR) 1.09, 95% confidence interval (CI) 1.05–1.14, *p* < 0.0001), and RC also had a positive correlation with the risk of MAFLD (OR 1.47, 95% CI 1.14–1.89, *p* < 0.0001). However, an inverse correlation was observed between RC and high socioeconomic status.

**Table 3:** Distribution of baseline characteristics ccross RC Quartiles.

Characteristics	RC levels quartiles, mg/dl				*P-value*
	Q1	Q2	Q3	Q4	
	2–12 (n = 1351, 27.76%)	13–18 (n = 1144, 23.51%)	19–27 (n = 1195, 24.56%)	28–99 (n = 1176, 24.17%)	
Age					< 0.0001
18–34	263 (43.77)	141 (33.41)	111 (23.65)	96 (17.56)	
35–49	125 (21.08)	114 (20.33)	119 (23.33)	148 (28.59)	
50–64	124 (22.39)	170 (28.86)	192 (25.74)	200 (31.95)	
> = 65	121 (12.75)	136 (17.40)	178 (27.29)	149 (21.90)	
Sex					0.08
Female	367 (57.09)	311 (52.12)	300 (50.85)	272 (45.35)	
Male	266 (42.91)	250 (47.88)	300 (49.15)	321 (54.65)	
Ethnicity					< 0.001
White	193 (60.30)	182 (62.92)	199 (60.43)	226 (64.17)	
Black	221 (17.75)	145 (11.92)	107 (9.10)	61 (5.27)	
Hispanics/Latin Americans	58 (6.34)	78 (9.48)	105 (11.03)	113 (12.73)	
Asian	43 (6.21)	46 (5.83)	74 (8.70)	64 (6.23)	
Other	118 (9.40)	110 (9.85)	115 (10.75)	129 (11.60)	
Educational level					0.02
Less than High school	103 (9.04)	94 (9.22)	156 (16.54)	144 (13.75)	
High school graduate or GED	373 (58.70)	345 (59.24)	302 (58.45)	329 (61.35)	
Some college or above	157 (32.27)	122 (31.55)	142 (25.00)	120 (24.90)	
Poverty income ratio					0.36
< 1.30	177 (19.62)	146 (18.98)	177 (23.50)	177 (22.37)	
1.30–3.49	251 (35.65)	259 (38.87)	253 (39.74)	254 (38.28)	
> = 3.50	205 (44.73)	156 (42.15)	170 (36.76)	162 (39.35)	

n(%) for categorical variables.

**Table 4:** Laboratory parameters across RC Quartiles.

Variables	RC levels quartiles, mg/dl				*P-value*
	Q1	Q2	Q3	Q4	
	2–12 (n = 1351, 27.76%)	13–18 (n = 1144, 23.51%)	19–27 (n = 1195, 24.56%)	28–99 (n = 1176, 24.17%)	
Body mass index (kg/m^2^)	26.69 (25.66, 27.72)	29.09 (28.46, 29.72)	30.84 (29.91, 31.78)	31.32 (30.45, 32.19)	< 0.0001
Waist circumference (cm)	91.69 (89.39, 94.00)	98.95 (97.66, 100.25)	104.28 (101.85, 106.72)	105.95 (103.62, 108.27)	< 0.0001
Systolic blood pressure (mmHg)	116.84 (115.32, 118.36)	122.85 (121.51, 124.19)	126.87 (123.86, 129.87)	125.34 (123.78, 126.90)	< 0.0001
Diastolic blood pressure (mmHg)	69.04 (67.67, 70.40)	72.38 (71.11, 73.66)	73.87 (71.69, 76.05)	73.99 (73.00, 74.99)	< 0.0001
Fasting insulin (uU/ml)	8.66 (7.45, 9.87)	10.85 (9.85, 11.85)	16.10 (14.52, 17.68)	16.97 (15.09, 18.86)	< 0.0001
Plasma fasting glucose (mg/dl)	101.89 (100.25, 103.54)	103.94 (101.90, 105.99)	111.62 (109.14, 114.09)	122.62 (117.48, 127.75)	< 0.0001
HbA1c (%)	5.39 (5.34, 5.45)	5.49 (5.43, 5.55)	5.75 (5.65, 5.85)	6.01 (5.88, 6.14)	< 0.0001
Aspartate aminotransferase (U/L)	21.92 (19.83, 24.01)	21.59 (20.29, 22.88)	22.99 (21.45, 24.53)	21.63 (20.97, 22.30)	0.36
Alanine aminotransferase (U/L)	19.41 (16.91, 21.91)	21.40 (20.20, 22.59)	25.08 (23.11, 27.05)	25.44 (24.07, 26.82)	< 0.0001
Gamma–glutamyl transferase (U/L)	21.12 (18.41, 23.82)	26.76 (22.30, 31.21)	32.69 (29.85, 35.52)	34.80 (32.25, 37.34)	< 0.0001
Triglycerides (mg/dl)	46.74 (45.81, 47.68)	76.97 (76.02, 77.92)	112.26 (111.15, 113.36)	219.35 (204.02, 234.67)	< 0.0001
Total cholesterol (mg/dl)	169.54 (164.78, 174.29)	180.51 (173.90, 187.13)	190.02 (185.58, 194.46)	206.01 (201.35, 210.68)	< 0.0001
HDL (mg/dl)	62.39 (59.90, 64.89)	56.71 (54.78, 58.65)	51.82 (50.01, 53.62)	44.35 (43.06, 45.64)	< 0.0001
LDL (mg/dl)	97.84 (93.85, 101.84)	108.42 (102.82, 114.02)	115.74 (112.37, 119.12)	118.71 (113.96, 123.45)	< 0.0001
Albumin (g/dl)	4.12 (4.07, 4.17)	4.03 (3.98, 4.08)	4.00 (3.95, 4.04)	4.01 (3.97, 4.06)	0.002
HS-CRP (mg/l)	3.19 (2.24, 4.14)	3.91 (3.00, 4.82)	4.13 (3.70, 4.57)	4.35 (3.43, 5.28)	0.04
WBC (1000 cells/uL)	6.10 (5.84, 6.36)	6.76 (6.57, 6.96)	7.07 (6.81, 7.33)	7.60 (7.32, 7.88)	< 0.0001
Hemoglobin (g/dL)	14.00 (13.81, 14.18)	14.28 (14.11, 14.44)	14.35 (14.17, 14.53)	14.56 (14.38, 14.75)	< 0.001
Platelets (1000 cells/uL)	229.43 (221.05, 237.80)	240.60 (233.27, 247.93)	243.88 (236.90, 250.85)	244.96 (235.15, 254.77)	0.01
HOMA-IR	2.59 (1.96, 3.22)	2.92 (2.62, 3.21)	4.82 (4.04, 5.61)	5.58 (4.79, 6.37)	< 0.0001
HOMA-IR (quartile)					< 0.0001
0.12–1.58	47.78 (40.39, 55.17)	32.55 (25.12, 39.98)	18.50 (12.45, 24.55)	11.45 (7.09, 15.81)	
1.58–2.56	29.57 (24.63, 34.51)	28.57 (22.13, 35.00)	22.53 (16.97, 28.10)	24.04 (18.38, 29.69)	
2.56–4.59	13.01 (9.49, 16.53)	26.44 (20.60, 32.27)	29.85 (23.12, 36.58)	26.84 (23.63, 30.06)	
4.59–179.18	9.64 (4.43, 14.85)	12.45 (8.40, 16.49)	29.12 (24.20, 34.05)	37.67 (31.81, 43.53)	
VCTE measurements					
CAP (dB/m)	233.09 (227.85, 238.33)	253.97 (250.18, 257.76)	278.97 (273.01, 284.92)	290.88 (285.31, 296.46)	< 0.0001
LSM (kPa)	5.34 (5.07, 5.61)	5.31 (4.99, 5.63)	6.08 (5.52, 6.63)	6.23 (5.66, 6.80)	0.03
					< 0.0001
CAP < 285	85.78 (81.54, 90.02)	72.01 (68.81, 75.21)	53.74 (48.06, 59.42)	48.02 (42.31, 53.74)	
CAP ≥ 285/LSM < 8.6	8.69 (5.35, 12.03)	15.42 (11.16, 19.68)	22.68 (18.33, 27.03)	24.19 (18.97, 29.41)	
CAP ≥ 285/LSM ≥ 8.6	5.53 (3.16, 7.90)	12.57 (8.78, 16.36)	23.58 (17.94, 29.22)	27.79 (23.83, 31.76)	

Weighted mean or percentage with 95% confidence intervals for continuous variables.

*HbA1c* glycosylated hemoglobin, type A1c; *HDL* high-density lipoprotein cholesterol; *LDL* low-density lipoprotein cholesterol; *RC* remnant cholesterol; *HS-CRP* hypersensitive-c-reactive-protein; *WBC* white blood cells; *HOMA-IR* homeostasis model assessment-insulin resistance; *CAP* controlled attenuation parameter; *LSM* liver stiffness measure; *VCTE* vibration- controlled transient elastography.

**Table 5:** Relationship between RC (mg/dl) and HOMA-IR.

	Crude Model		Model 1		Model 2	
	OR (95% CI)	*P-value*	OR (95% CI)	*P-value*	OR (95% CI)	*P-value*
Age	1.02 (1.02, 1.03)	< 0.0001	1.02 (1.02, 1.03)	< 0.0001	1.02 (1.01, 1.02)	< 0.0001
Sex						
Female	1 (ref.)	–	1 (ref.)	–	1 (ref.)	–
Male	1.36 (1.07, 1.74)	0.02	1.38 (1.13, 1.70)	< 0.001	1.37 (1.13, 1.67)	< 0.001
Ethnicity						
White	1 (ref.)	–	1 (ref.)	–	1 (ref.)	–
Black	0.47 (0.33, 0.68)	< 0.001	0.40 (0.31, 0.52)	< 0.0001	0.38 (0.29, 0.50)	< 0.0001
Hispanics/Latin Americans	1.69 (1.11, 2.57)	0.03	1.52 (1.07, 2.16)	0.02	1.63 (1.13, 2.34)	0.01
Asian	1.07 (0.70, 1.65)	0.76	1.34 (0.90, 1.98)	0.15	1.40 (0.94, 2.10)	0.10
Other	1.10 (0.81, 1.50)	0.55	1.08 (0.82, 1.44)	0.57	1.24 (0.92, 1.67)	0.15
Educational level						
High school or less	1 (ref.)	–	1 (ref.)	–	1 (ref.)	–
High school graduate or GED	0.70 (0.49, 0.99)	0.07	0.79 (0.61, 1.02)	0.07	0.78 (0.57, 1.07)	0.12
Some college or above	0.58 (0.40, 0.82)	0.01	0.68 (0.51, 0.91)	0.01	0.70 (0.51, 0.96)	0.03
Poverty income ratio						
< 1.30	1 (ref.)	–	1 (ref.)	–	1 (ref.)	–
1.30–3.49	0.99 (0.74, 1.32)	0.95	0.97 (0.76, 1.25)	0.83	0.95 (0.74, 1.21)	0.65
> = 3.50	0.80 (0.56, 1.14)	0.24	0.75 (0.56, 1.00)	0.05	0.72 (0.54, 0.95)	0.02
Non-MAFLD	1 (ref.)		1 (ref.)		1 (ref.)	
MAFLD	3.89 (3.00, 5.04)	< 0.0001	1.62 (1.28, 2.05)	< 0.0001	1.47 (1.14, 1.89)	< 0.001
HOMA-IR	1.17 (0.99, 1.38)	0.08	1.19 (1.14, 1.24)	< 0.0001	1.09 (1.05, 1.14)	< 0.0001
Quartiles						
0.12–1.58	1 (ref.)	–	1 (ref.)	–	1 (ref.)	
1.58–2.56	1.96 (1.32, 2.89)	0.01	1.68 (1.31, 2.15)	< 0.0001	1.53 (1.19, 1.98)	< 0.001
2.56–4.59	4.92 (3.24, 7.47)	< 0.0001	3.73 (2.79, 4.99)	< 0.0001	3.17 (2.33, 4.32)	< 0.0001
4.59–179.18	6.38 (3.22, 12.63)	< 0.001	5.48 (3.87, 7.74)	< 0.0001	4.41 (3.01, 6.45)	< 0.0001

Crude Model: adjust for none.

Model 1: adjust for age, sex, ethnicity, educational level, poverty income ratio.

Model 2: adjust for age, sex, ethnicity, educational level, poverty income ratio, BMI, waist circumference, alcohol taking, smoking, systolic blood pressure, diastolic blood pressure.

### Receiver operating characteristic analysis

**Figure 2:**
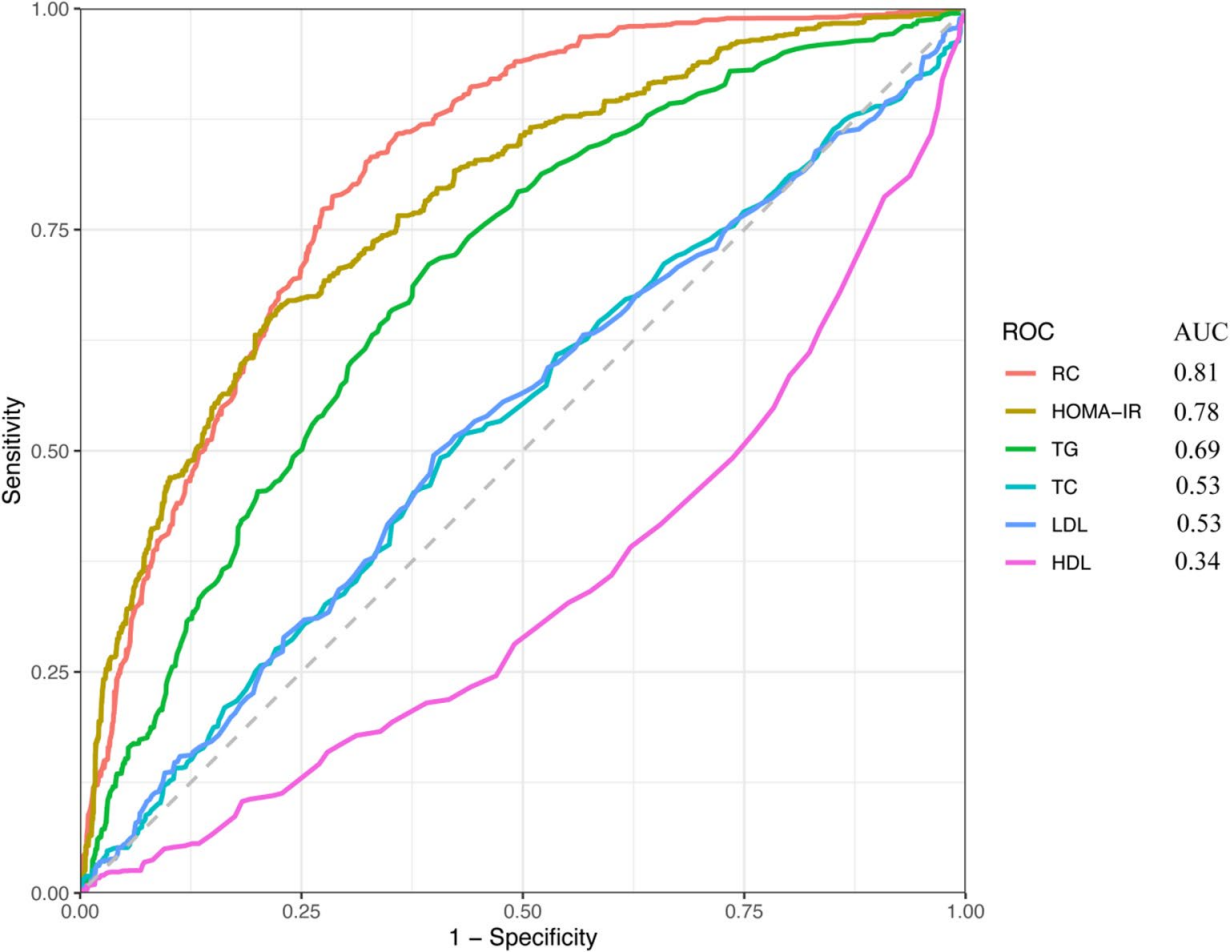
ROC curve analysis of MAFLD-related lipid parameters and HOMA-IR. ROC, receiver operating characteristic; RC, remnant cholesterol; HOMA-IR, homeostasis model assessment-insulin resistance; HDL, high-density lipoprotein cholesterol; LDL, low-density lipoprotein cholesterol; TG, triglycerides; TC, total cholesterol.

As shown in Fig. 2, the predictive value for MAFLD, which was highest for RC, followed by HOMA-IR, was better than TG, TC, LDL, and HDL.

### Association between RC and HOMA-IR

**Figure 3:**
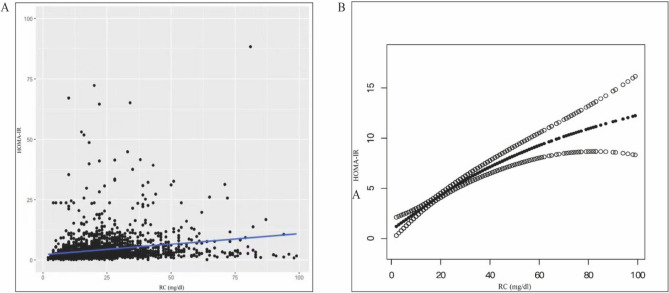
Relationship between RC and HOMA-IR. (**A**) Each black dot represents a sample. (**B**) Solid black line represents the smooth curve fit variables. The black dotted line shows a 95% confidence interval (CI) for the fit. They adjusted for age, sex, ethnicity, educational level, poverty income ratio, BMI, waist circumference, alcohol taking, smoking, systolic blood pressure, diastolic blood pressure.

We also used weighted generalized models and smoothing curve fitting to determine the association between RC and HOMA-IR (Fig. 3A,B). We further examined the threshold effect of RC on HOMA-IR using a nonlinear model (log-likelihood ratio = 0.003, Supplementary Table 1). For participants with MAFLD, when RC increased by 1 mg/dl, HOMA-IR of HOMA-IR increased by 0.17 (RC < 30 mg/dl). This result is consistent with the curve-fitting plots.

### Subgroups

Supplementary Fig. 1 reveals significant variations in MAFLD risk across RC levels, further stratified by HOMA-IR and BMI. This underscores the differential impact of RC, HOMA-IR, and BMI on MAFLD risk among participants. The higher the HOMA-IR level, the higher the risk of RC-related MAFLD (OR 1.95 per SD increase for the highest HOMA-IR level vs. OR 1.02 per SD increase for the lowest HOMA-IR level, P-interaction = 0.04). For BMI subgroups, the risk of RC-related MAFLD was higher in underweight people (OR 2.57 per SD increase with underweight people vs. OR 1.34 per SD increase with obesity, P-interaction = 0.001).

### Association between RC and MAFLD vs NAFLD

As shown in Supplementary Table 2, the association was significantly stronger for increased levels of RC with MAFLD (OR 4.11, 95% CI 0.45–7.77, *p* < 0.0001) than NAFLD (OR 2.1, 95% CI − 0.52–4.71, *p* < 0.0001).

## Discussion

The current study offers a comprehensive exploration of the prevalence, clinical characteristics, and the influence of RC on MAFLD within a multiethnic population in the United States. This investigation is the first to establish a positive correlation between RC and HOMA-IR in individuals with MAFLD. The prevalence of MAFLD was found to be approximately 44.7%, predominantly in middle-aged and older men. Compared to non-MAFLD patients, those with MAFLD exhibited higher levels of metabolic, inflammatory, lipid, and hepatic biomarkers and a higher prevalence of CSF. Among these, RC and HOMA-IR increased linearly with MAFLD prevalence.

The established role of LDL-C in cardiovascular disease is well-documented, with lower levels of LDL-C being associated with a reduced risk of cardiovascular events^24^. However, even with optimal control of LDL-C and management of other cardiovascular risk factors, a residual risk persists. Recent studies have highlighted that elevated levels of RC, found in triglyceride-rich lipoproteins, may contribute to this remaining cardiovascular risk^25–27^. RC has the capability to permeate arterial walls, potentially leading to atherosclerosis, foam cell formation, and low-grade inflammation. These conditions are exacerbated when RC is overexpressed in plasma due to factors such as excessive calorie consumption, obesity, diabetes, and genetic variations affecting lipoprotein metabolism^28,29^.

Recent research has shown that the redefined MAFLD is linked to increased probabilities of fatalities from all-cause and specific causes, indicating the urgent need for the early identification of high-risk individuals^30^. Furthermore, MAFLD development has been significantly influenced by disturbances in lipoprotein metabolism. Epidemiological studies have indicated that RC may serve as an independent predictor of long-term mortality in patients with MAFLD^31^. Our findings further reinforce this perspective, clearly demonstrating that higher levels of RC are associated with an increased risk of liver fibrosis in MAFLD patients. This association was consistent between patients with and without CSF, suggesting elevated RC levels may predict progression of liver fibrosis in MAFLD. The significant liner trend emphasizes the strength of the association between RC and liver fibrosis risk in MAFLD. Monitoring RC levels could aid fibrosis risk assessment in MAFLD patients. These findings point to a potential role of RC in the pathophysiology of liver fibrosis in MAFLD. Higher RC levels were associated with a higher risk of MAFLD, regardless of sex, age, and ethnicity. Notably, having a college degree or higher and having a PIR ≥ 3.5% of the poverty level were both negatively associated with RC for patients with MAFLD. A previous study revealed that increased obesity and socioeconomic status, as gauged by educational status, are causally associated with biological risk factors (lipids)^32^. This suggests that changing socioeconomic status is crucial for causing and controlling MAFLD.

Kessoku et al.^33^ examined 1365 biopsy-proven NAFLD cases included in the JSG-NAFLD database, revealing that HOMA-IR significantly increased with the stage of hepatic fibrosis. This underscores the progressive nature of IR in relation to liver disease severity. Similarly, Ballestri et al.^34^ demonstrated HOMA-IR as an independent predictor of progressive hepatic fibrosis in patients with biopsy-proven NAFLD, further substantiating the critical role of IR in the pathogenesis of liver fibrosis. Additionally, a HOMA-IR value of ≥ 2.90 was found to be an independent predictor of advanced fibrosis in nondiabetic NAFLD patients, suggesting a direct pathway where IR may activate hepatic stellate cells (HSCs)^35^. This finding is pivotal as it highlights the direct impact of metabolic dysfunction at the cellular level within the liver. Furthermore, hypertriglyceridemia, a key component of insulin resistance syndrome, has been linked to these hepatic changes. Ohnishi et al.^36^ observed a significant positive correlation between HOMA-IR and remnant-like particle cholesterol in Japanese individuals, aligning with the global understanding of dyslipidemia in metabolic diseases. This study’s results supported a positive correlation between higher RC and higher HOMA-IR in MAFLD patients, consistent with a previous study on RC. The study also observed that when the RC level increased by 1 mg/dl, HOMA-IR increased by 0.17 (RC < 30 mg/dl). These results underscore the role of IR in MAFLD. The positive correlation between HOMA-IR and RC levels in MAFLD patients supports the hypothesis that IR may exacerbate lipid dysregulation, contributing to MAFLD progression. This is in line with studies that have identified IR as a key driver of hepatic steatosis and inflammation in MAFLD.

This study highlights significant variations in the risk of MAFLD associated with RC across different BMI categories. Intriguingly, our findings indicate that individuals with a lower BMI may also be at a heightened risk of developing RC-related MAFLD. While it is well recognized that a higher BMI is commonly associated with an increased risk of cardiometabolic diseases, recent research has also identified a metabolically unhealthy, lean phenotype, albeit less prevalent. This underscores the complexity of metabolic disease risk, which is not solely confined to obesity^37^. Further exploration into the relationship between RC levels and MAFLD, particularly in varying BMI contexts, could open up new avenues for treatment strategies focusing on RC regulation. And in this study, the higher levels of RC could significantly increase the risk for MAFLD compared with NAFLD.

Despite these significant findings, this study has several limitations. Due to insufficient serial data on the relationship between RC and IR in the context of MAFLD, we couldn't evaluate their longitudinal variations. Moreover, while several covariates were controlled for, the potential for unaddressed and unmeasured confounding influences remains. Additionally, the representativeness of the study's sample across diverse ethnic and socioeconomic groups is limited, potentially affecting the generalizability of the results. Future research should focus on mechanistic studies to elucidate the underlying biochemical pathways of these associations. Interventional trials are also needed to assess the effectiveness of targeting RC and IR in managing MAFLD. Furthermore, exploring the impact of unaccounted confounders like genetic factors and lifestyle habits and conducting studies on dietary and pharmacological interventions could provide more targeted approaches for MAFLD management.

In conclusion, this study establishes a significant positive correlation between RC and the HOMA-IR in adults, shedding light on the identification and severity assessment of MAFLD. These findings suggest that RC can serve as an accessible, cost-effective, and reliable indicator for assessing MAFLD risk. Importantly, RC can aid clinicians in early identification of high-risk individuals, facilitating timely diagnostic confirmation through ultrasound and the initiation of early interventions, ultimately contributing to improved patient outcomes and potentially reducing MAFLD prevalence.

### Supplementary Information

Supplementary Information.

## Data Availability

Data can be downloaded from NHANES database (https://wwwn.cdc.gov/nchs/nhanes/continuousnhanes/default.aspx?BeginYear=2017).
