# Supplementary material for: Association between remnant cholesterol and insulin resistance levels in patients with metabolic-associated fatty liver disease
Source: Sci Rep. 2024 Feb 26;14:4596. doi: 10.1038/s41598-024-55282-4 (PMC10897142; doi:10.1038/s41598-024-55282-4)
Supplement: Supplementary file 1 — Supplementary Information. [file 41598_2024_55282_MOESM1_ESM.docx]

Supplementary Table 1 Threshold effect analysis of RC on HOMA-IR using the two-piecewise linear regression model

|  | Adjusted β(95% CI) | *P-value* |
| --- | --- | --- |
| Fitting by the standard linear model | 0.12(0.10,0.14) | <0.001 |
| Fitting by the two-piecewise linear model |  |  |
| Inflection point | 30mg/dl |  |
| RC<30mg/dl | 0.17( 0.13, 0.22) | <0.001 |
| RC>30mg/dl | -0.05( -0.14, 0.03) | 0.19 |
| Log-likelihood ratio | 0.003 |  |

Age, sex, ethnicity, educational level, poverty income ratio, BMI, waist circumference, alcohol taking, smoking, systolic blood pressure, diastolic blood pressure were adjusted.

Supplementary Table 2 Relationship between RC (mg/dl) and MAFLD vs NAFLD

| character | Q1 | Q2 OR(95% CI) | *p-value* | Q3 OR(95% CI) | *p-value* | Q4 OR(95% CI) | *p-value* | *p for trend* |
| --- | --- | --- | --- | --- | --- | --- | --- | --- |
| MAFLD-  NAFLD- | ref | 0.37(-0.29, 1.04) | 0.25 | 1.53( 0.18, 2.88) | 0.03 | 1.65( 0.65, 2.64) | 0.003 | 0.002 |
| MAFLD+  NAFLD- | ref | 0.99(-0.08, 2.07) | 0.07 | 2.37( 0.76, 3.98) | 0.01 | 4.11( 0.45, 7.77) | <0.0001 | <0.0001 |
| MAFLD-  NAFLD+ | ref | 0.62( 0.17,1.07) | 0.01 | 1.71( 1.16,2.26) | <0.0001 | 2.1(-0.52,4.71) | <0.0001 | <0.0001 |
| MAFLD+  NAFLD+ | ref | 0.58( 0.18,0.97) | 0.07 | 1.66( 1.20,2.13) | 0.01 | 2.01( 1.59, 2.44) | <0.0001 | <0.0001 |

Age, sex, ethnicity, educational level, poverty income ratio, BMI, waist circumference, alcohol taking, smoking, systolic blood pressure, diastolic blood pressure were adjusted.


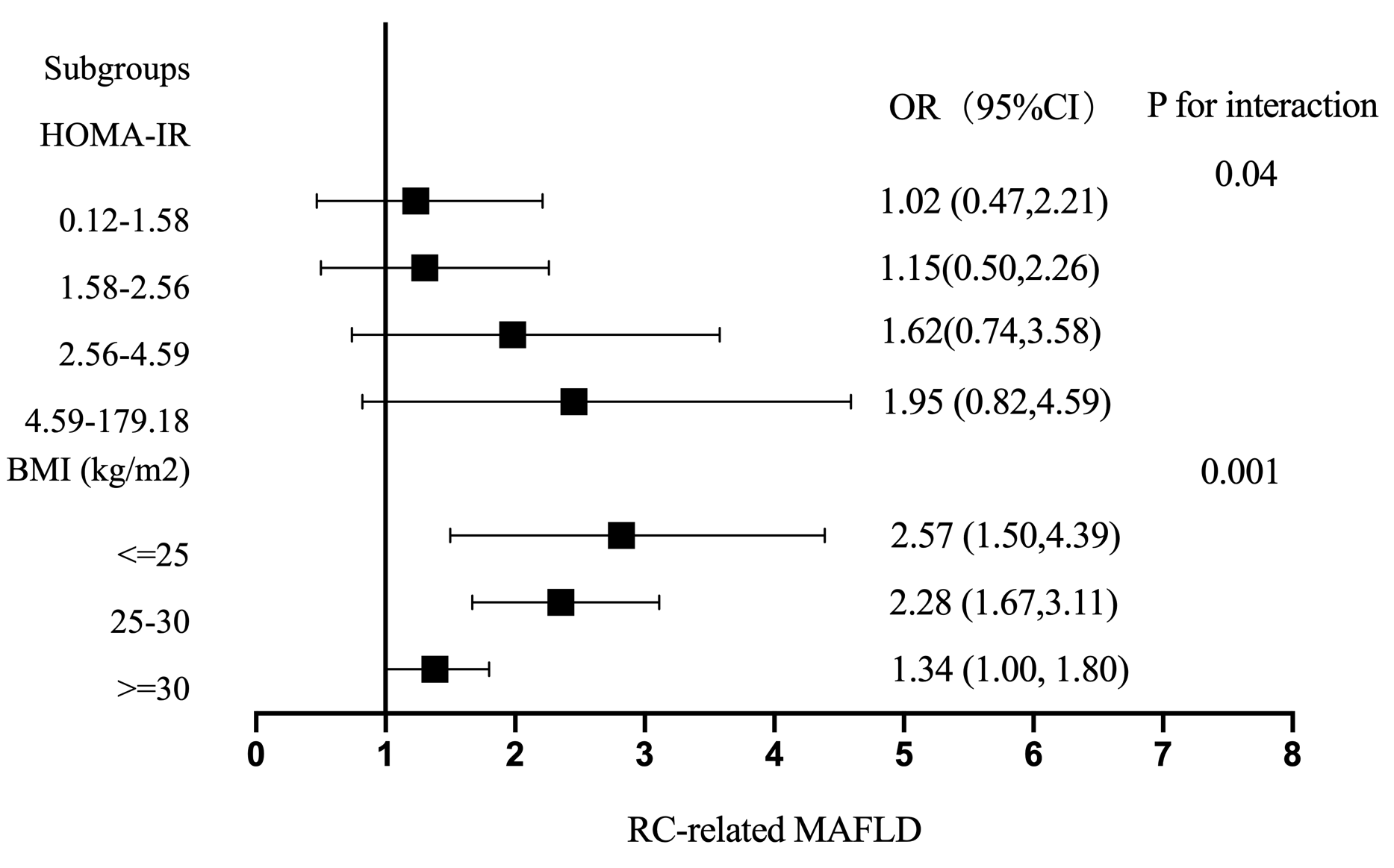


Supplementary Figure 1 Stratified associations between RC and MAFLD by HOMA-IR, and BMI. OR, odds rations; CI, confidence interval; BMI, body mass index. Age, sex, ethnicity, educational level, poverty income ratio, waist circumference, alcohol taking, smoking, systolic blood pressure, diastolic blood pressure were adjusted.
